# Tourette syndrome research highlights from 2022

**DOI:** 10.12688/f1000research.135702.2

**Published:** 2023-10-23

**Authors:** Andreas Hartmann, Per Andrén, Cyril Atkinson-Clément, Virginie Czernecki, Cécile Delorme, Nanette Marinette Monique Debes, Kirsten Müller-Vahl, Peristera Paschou, Natalia Szejko, Apostolia Topaloudi, Keisuke Ueda, Kevin J. Black

**Affiliations:** 1 Department of Neurology, Pitié-Salpêtrière Hospital, Assistance Publique - Hopitaux de Paris, Paris, Île-de-France, 75013, France; 2 Department of Psychology, University of Lund, Lund, Sweden, Sweden; 3 School of Medicine, University of Nottingham, Nottingham, England, UK; 4 Herlev University Hospital, Department of Child Neurology, University of Copenhagen, Copenhagen, Denmark; 5 Department of Psychiatry, University of Hannover, Hannover, Germany; 6 College of Science, Purdue University, West Lafayette, Indiana, USA; 7 Department of Neurology, University of Calgary, Calgary, Alberta, Canada; 8 Department of Psychiatry, Washington University School of Medicine, Saint Louis, Missouri, 63110-1010, USA

**Keywords:** Tourette, tics, annual review

## Abstract

This is the ninth yearly article in the Tourette Syndrome Research Highlights series, summarizing selected research reports from 2022 relevant to Tourette syndrome. The authors briefly summarize reports they consider most important or interesting.

## Introduction

This article is meant to disseminate scientific progress on Gilles de la Tourette Syndrome (TS) that appeared in the year 2022, summarizing research reports the authors judged important or interesting.

## Methods

We searched PubMed using the search strategy (“Tic Disorders”[MeSH] OR Tourette NOT Tourette [AU] NOT Tourette [COIS]) AND 2022[PDAT] NOT 1950:2021[PDAT]. On 09 January 2023 this search returned 216 citations, available at this link. Colleagues also recommended articles, and we attended selected medical conferences. We selected material for this review subjectively, guided by our judgment of possible future impact on the field.

## Results

### Phenomenology and natural history

**Definition and phenomenology**

On blinded video review, the frequency of “extra” (non-goal-directed) movements was more than three times higher in patients with a tic disorder than in people without a tic disorder, vocalizations were greater than 22 times more frequent, and obsessive-compulsive and depressive symptoms were much more common ( Bartha *et al*. 2023). However, the occurrence and frequency of “extra” movements overlapped between those with and without a tic disorder; therefore, the authors concluded that surplus movements *per se* are not sufficient to diagnose tics. The repetitive character, timing pattern, and associated features of tics such as premonitory urges are necessary too. These results will not surprise tic clinicians but highlight the difficulties in attempting to diagnose and count tics by artificial intelligence analysis of video recordings.

Nilles and colleagues presented data from a consecutive series of 200 youth with primary tic disorders at a referral center starting in 2017 ( Nilles *et al*. 2023). Females had more severe motor tics and impairment from tics than did males, and tic severity increased with age.

Bazaibal-Carvallo and colleagues published a study about self-injurious behaviors (SIB) in TS ( Baizabal-Carvallo *et al*. 2022). The authors included 201 patients with TS and 34 (16.9%) of them had comorbid SIB. The majority of these patients experienced self-directed SIB (11.4%), while only 3.5% of participants also experienced aggression towards others and only 2% had what the authors denominated “tic-related SIB”, i.e., clinical injury secondary to severe tics. In this study, the authors compared in detail the distribution of different tics in patients with SIB and without using a univariate model, and concluded that individuals with SIB are more inclined to have tics involving shoulder, trunk, arm, as well as dystonic tics, complex motor tics, copropraxia, complex phonic tics, a higher number of phonic tics, coprolalia and OCD. In multivariate analysis, SIB was found to be associated with complex motor tics, obsessive-compulsive symptoms and greater tic severity. Interestingly enough, patients with SIB were also more likely to have been selected for deep brain stimulation (DBS).

A huge electronic medical records database showed evidence that tic disorders in children are associated with atopic disorders ( Hakimi *et al*. 2022). Rates of atopy were higher in children who had taken a methylphenidate-class drug or an α _2_ agonist; this result could implicate the medications, or it could reflect higher severity of tics leading to treatment.

**Assessment**

A nearly universally wished-for advance in TS research would be a reliable automated tic detector. One such was described at the European Society for the Study of Tourette Syndrome (ESSTS) meeting in June 2022 in Lausanne, Switzerland. First, a neural network detected a set of facial landmarks to reduce the dimensionality of the data from a video stream of the person’s face ( Loewenstern *et al*. 2022). A complex processing stream used multiple convolutional neural networks to identify salient spatial features mapping to tics, and a different form of neural network to identify temporal signatures of tics. The algorithm detected over 90% of tics from video captured by a smartphone camera. However, the group had not yet performed out-of-sample generalization testing.

**Epidemiology**

Several studies addressed the prevalence and incidence of TS and chronic tic disorders. Using 2016–2017 National (U.S.) Survey of Children’s Health data, parents reported that 0.3% of their children and adolescents between 6 and 17 years ever had been diagnosed with TS ( Charania *et al*. 2022). A systematic review and meta-analysis showed a global prevalence of TS of 0.5–0.6% including adults and of 0.7% in children and adolescents ( Jafari *et al*. 2022). In Asia, the National Taiwan Insurance Research Database was used to estimate incidence and prevalence of TS and chronic tic disorders (TS/CTD) from 2007–2015 ( Chou *et al*. 2022). The prevalence of TS/CTD increased during this period. Annual incidence rates of TS/CTD increased in childhood and adolescence but decreased in adulthood. In China, the prevalence of tic disorders in school students between 6 and 16 years was reported as 1.37%, lower than almost every other recent study ( Yan *et al*. 2022). TS and OCD were highly comorbid.

A report from the U. S. Centers for Disease Control and Prevention (CDC) attempted to synthesize the varied data on prevalence of persistent (chronic) tic disorders in the US ( Tinker *et al*. 2022). They estimate that 350,000–400,000 children and adults have TS, and about a million more have persistent motor or vocal tic disorder, but they conclude that new data with more accurate methods is required to be more confident in the results.

**Prognosis and natural history**

Ricketts and colleagues evaluated childhood predictors of adult tic severity and impairment using a multiple linear regression analysis and found that childhood severity of tics and female sex ( *p* > 0.002) are important predictors for tic severity and impairment in adulthood ( Ricketts *et al*. 2022d).

Using an online survey, 351 adult males with TS completed questionnaires about adverse childhood experiences (ACEs) and a lifetime version of the Yale Global Tic Severity Scale ( Yang *et al*. 2022). An association between ACEs and higher lifetime tic severity and impairment was found. TS genetic risk may moderate the association between ACE score and tic impairment.

Openneer and colleagues conducted a subanalysis of the European Multicentre Tics in Children Study (EMTICS) to evaluate tic predictors in a prospective study ( Openneer *et al*. 2022). Children with tic onset were more frequently male, had higher baseline severity of conduct problems, autism spectrum disorder (ASD) symptoms, compulsions and emotional problems compared to children without tic onset. Interestingly, there were differences in tic predictors between sexes: while conduct and ASD problems were male-specific predictors, severity of compulsions and oppositional and emotional problems were female-specific predictors.

A very complete review and overview of Tourettic OCD (TOCD) was published by Katz and colleagues ( Katz *et al*. 2022). This phenotype, well-known to all clinicians working with TS patients, consists of touching behavior, symmetry concerns, and thoughts of exactness. The authors feel that the core of the phenomenon is a need to repeat complex tics until “just right,” without cognitive or imaginal obsessions. The authors offer arguments why TOCD might be a condition distinct from both TS and OCD and advocate its inclusion as a separate entity in future editions of the DSM. The authors suggest studies that may allow us to delimit TOCD clearly from TS and OCD ( Robins and Guze 1970), though at this time some needed evidence is lacking.

Li and colleagues reported on children with refractory TS ( Li *et al*. 2022). Pediatric refractory TS was characterized by earlier age of onset, longer disease duration, lower IQ, higher prevalence of premonitory urges (PU) and higher prevalence of psychiatric comorbidities.

**Sensory phenomena and premonitory urge**

Essing and colleagues published an important study regarding the location of premonitory urges (PU) in patients with tics ( Essing *et al*. 2022), updating the conference presentation we reviewed previously ( Jakubovski *et al*. 2018; Rose *et al*. 2019). This was an online study in 291 adults with tic disorders in which participants self-reported about different characteristics of PU. The authors found that PU were located in the same body part or in direct proximity to the part of the body where tic was located. Most frequently, PU were found in the area of the face and the head (62%). Complex tics were more frequently preceded by PU than simple tics but there were no differences regarding report of PU for motor *vs.* vocal tics. Usually, PU were localized in the front rather than the back of the body (73% vs. 27%), but there were no differences between the right and left sides.

Langelage and colleagues examined urge-tic association in 25 children and adolescents with TS ( Langelage *et al*. 2022). The authors were interested in urge-tic associations, including inter-individual differences, and correlation with clinical measures, and compared their findings with a sample of adults. At the group level, the authors found positive associations between urges and tics, but this was not confirmed in the individual level data since fewer than half of participants showed a positive association between urge and tic, and two participants had reverse (negative) association. Similarly to previous reports, associations between urge levels and tic intensity were less pronounced in children and adolescents than adults with TS.

He and colleagues tested the contribution of brain γ-aminobutyric acid (GABA) and glutamate levels in the right primary sensorimotor cortex (SM1), supplementary motor area (SMA), and insular cortex (insula) to tic and urge severity in children with TS ( He *et al*. 2022). Overall, they demonstrated involvement of GABAergic neurotransmission within the SMA in the experience of PU in children with TS.

Isaacs and colleagues published two reports on sensory phenomena in adults with chronic tic disorders (CTD). The first examined sensory hypersensitivity (SH) in over 200 adults with CTD, OCD or neither ( Isaacs *et al*. 2022). Both patient groups had much higher self-reported SH than did healthy controls, but tics did not confer additional sensitivity. Across all three groups, SH was associated with OCD and attention deficit hyperactivity disorder (ADHD) symptoms but not with tic severity. The second report investigated interoception in adults with or without CTD using a published self-report measure ( Narapareddy *et al*. 2022). Results indicated that adults with CTD are more aware of their internal body state and have higher self-reported anxious somatization scores, but these results are explained primarily by female sex and OCD symptoms, not tics. They identified a collection of symptoms from the interoception questionnaire that correlated with premonitory urge severity.

**Comorbidities**

Araújo and colleagues examined the role of ADHD and OCD in tic severity during the COVID-19 pandemic in Brazilian and Portuguese patients with TS and found that approximately half of the patients experienced worsening of tic severity ( Araújo *et al*. 2022). They suggest patients with comorbidity might be more susceptible to the effects of the pandemic.

Food difficulties—among others, greater food responsiveness and emotional overeating—were shown to be more common in children with TS than previously reported ( Smith *et al*. 2021).

The experience of tic-related pain and use of pain management was assessed in an online survey answered by 188 adults with self-reported tics ( Taylor *et al*. 2022). Tic-related pain was shown to have a significant physical and psychological impact and was important to be addressed in the long-term management of tic disorders. A similar study was conducted in Poland ( Małek 2022) but this time in a pediatric population. The authors included 40 children with TS and 57 parents of children with TS, as they wanted to collect information about the perspective of children and parents on this topic. For tic assessment the authors used the YGTSS, while pain severity, localization and coping strategies were assessed with the Pediatric Pain Questionnaire and Pediatric Pain Coping Inventory, which were administered both to children and parents. Pain was reported by 60% of children with TS and 72% of parents confirmed that their children sometimes suffered from pain. The most common sites of pain were the cervical region, throat, shoulder, ocular region, and joints. Contrary to expectations, no correlation was found between tic severity and pain. Consistency was observed between the reports of children and their parents in coping with pain.

Ricketts and colleagues published an important article about sleep disorders and the use of sleep medication, nighttime tics and pattern of sleep in patients with tics ( Ricketts *et al*. 2022a). In this study, 125 adults with tics completed an internet survey in which they rated sleep history and sleep chronotype (i.e., the natural inclination of your body to sleep at a certain time) as well as the severity of tics and psychiatric comorbidities. The most frequently reported sleep-related disorders in a population of patients with tic disorders were bruxism, insomnia, and tic-related difficulty falling asleep. Sleep problems correlated with impairment, obsessive-compulsive symptoms, and emotional regulation problems. Interestingly enough, eveningness related to tic severity. Therefore, the authors hypothesized that interventions to advance chronotype may help with tic improvement. The same group of authors examined another aspect of sleep disorders in the group of patients with tics ( Ricketts *et al*. 2022c). This study included 114 children with TS, and the authors compared those with sleep disorders (n = 32) to those who have no problems with sleep (n = 82). Children with TS and sleep disorder were from households with lower parental education and at higher risk of poverty. They also were more frequently diagnosed with comorbidities such as OCD, ODD, ADHD and ASD and were more frequently prescribed anti-tic medication. In line with these findings, children with TS and sleep disorder had more severe tics, tic-related impairment and more severe ADHD symptoms.

A case–control study including 271 children with tic disorder and 271 controls revealed that children with tic disorders had increased risks for sleep disturbances as measured with the Children’s Sleep Habits Questionnaire ( Mi *et al*. 2022). Sleep disturbances included among others bedtime resistance, sleep onset delay, sleep anxiety, night waking, and daytime sleepiness. The presence of comorbid ADHD increased the risk for sleep disturbances.

Jiménez-Jiménez and colleagues published results of a register-based cohort study to estimate the prevalence of insomnia in Swedish patients with tics ( Jiménez-Jiménez *et al*. 2022). Individuals with tics had a prevalence of insomnia of 32.2% in comparison to 13.7% in the general population, and this difference was statistically significant. Importantly, this association was independent from somatic disorders, familial factors, or psychiatric comorbidities, although familial factors, neurodevelopmental comorbidities, and ADHD/ADHD medication may explain part of the association.

Nail biting (onychophagia) is common in unselected children but has also been included in descriptions of complex tics. A report from Taiwan examines prevalence of nail biting in over 2000 children, including 765 with a primary tic disorder, and finds that nail biting is very common in TS (56.6%) and provisional tic disorder (27.4%)—much more common than in controls (15.0%)—and begins prior to onset of definite tics ( Hsueh and Chen 2022).

Vermillion and colleagues compared responses on the Youth Risk Behavior Surveillance System (YRBS) in youth with TS and healthy controls and did not show any differences between these groups regarding the profile of risky behaviors ( Vermilion *et al*. 2022).

Cui and colleagues reported on the emotional and behavioral profile of children with TS in China, using the Child Behavior Checklist (CBCL), and compared this profile with those of sex-matched healthy controls and groups with ADHD, OCD and depression ( Cui *et al*. 2022a). No association was found between the eight factors of the CBCL and motor tics, vocal tics or tic severity assessed by the YGTSS. Nevertheless, there was a positive association between the impairment scale of the YGTSS and thought problems as well as rule-breaking behavior. Contrary to expectation, children with GTS showed a similar profile of CBCL to the children with depression, but not ADHD or OCD.

Tessier and colleagues compared the design fluency profile of children with TS with matched healthy controls ( Tessier *et al*. 2022). Children with TS did not show general executive dysfunction in comparison to their peers.

Another study investigated how adults with TS make decisions in two different contexts ( Atkinson-Clement *et al.* 2022a): one involving choices between two options under ambiguity (i.e., when the probabilities of outcomes for each option are not fully known), and the other under risk (i.e., when the probabilities of outcomes are known, but there is a possibility of loss). This study was primarily motivated by the observation that individuals with TS are more prone to engaging in risky behaviors in their everyday lives. Interestingly, they discovered that TS was not associated with any abnormal decision-making processes. In contrast, they observed that ADHD and OCD were more pertinent, particularly in terms of distorting the probability of outcomes. Altogether, they suggested that the risk propensity observed in real-life could be not directly related to TS.

**Functional tic-like behaviors**

Fremer and colleagues examined 32 patients with functional tic-like behaviors (FTLBs) reflecting those found on social media, using both operationalized psychiatric diagnosis and a psychodynamic focus ( Fremer *et al*. 2022). Symptoms typically started abruptly at the mean age of 19 years and gradually deteriorated. As assessed by the Operationalized Psychodynamic Diagnostic System, a widely used and well-established instrument in psychodynamic psychotherapy that aims to explore underlying psychopathology, in all patients, current psychological stressors, unconscious intrapsychic conflicts (e.g., dependency and autonomy), and/or structural deficits (e.g., lack of certain basic mental abilities such as affect tolerance or regulation of self-esteem) were identified. Nearly all patients (94%) also suffered from other psychiatric symptoms. The authors concluded that pre-existing abnormalities in social behavior and psychiatric symptoms, but also TS in combination with time-related psychological stressors, unconscious intrapsychic conflicts, and structural deficits predispose to development of these symptoms.

Han and colleagues reported the prevalence and clinical characteristics of children with functional tic-like behaviors detected during the COVID-19 pandemic based on an analysis from a single center ( Han *et al*. 2022). There was a significant increase in the percentage of functional tic-like behaviors in 2020 and 2021. In line with other studies, differences between patients with functional tics included several distinguishing features: predominance of females, later age of onset and higher rates of anxiety and depression. Patients with FTLBs also more frequently demonstrated coprolalia-like behaviors, complex phrases, self-injurious behaviors, higher rates of hospitalizations and school absenteeism.

Trau and colleagues reviewed charts of 198 patients with tic-like phenomena and propose diagnostic criteria to separate those with typical tics, those with clinically diagnosed functional tics (FT), and those with a past typical tic presentation complicated by a fulminant worsening ( Trau *et al*. 2022). Only the presence of rostrocaudal progression and increased obsessive-compulsive behaviors were significantly different between patients with new-onset FT and those with functional worsening of a previous tic disorder. Results also showed that age at tic onset was not a contributing factor for group differentiation. Many patients with FT were not exposed to videos depicting tics on social media. Similarly, the Calgary group tested which symptoms differed between 41 children or teens with FTLBs and 195 with typical primary tic disorders ( Nilles *et al*. 2022b). The specific symptoms that best discriminated the two groups included copropraxia, coprolalia, popping or whistling noises, simple head movements, and self-injurious actions.

A group of experts gathered by the Tourette Association of America ( Malaty *et al*. 2022) developed a database from the recent spate of articles on FTLBs, including all the features thought to distinguish FTLBs from typical tic disorders. They summarized these reports and offered recommendations for diagnosing FTLB. The authors found some features to provide better evidence than others for differential diagnosis but emphasize that at this time the diagnosis generally needs to include several characteristics. Another review came from the Calgary group ( Amorelli *et al*. 2022).

Arbuckle and colleagues presented new data from the Washington University New Tics study on clinical features of children examined on average four months after tic onset, who would go on to be diagnosed with TS/CTD ( Arbuckle *et al*. 2022). They compare these characteristics to those described in 17 published reports on functional tic-like symptoms (FND-tic), whose initial evaluation also tends to be a few months after symptoms begin. Stark differences in presentation distinguish the FND-tic patients from typical PTD. Symptoms that best distinguished the groups in terms of positive or negative predictive value (PPV or NVP) included movements or vocalizations that were dramatically worse in the presence of others *vs.* alone, coprophenomena at presentation, symptoms that dramatically and persistently disrupt the person’s intended actions or communications, and “tic attacks.”

Howlett and colleagues investigated the prognosis of functional tic-like behaviors in 20 adolescents and nine adults ( Howlett *et al*. 2022). The authors found in this prospective study that adolescents with FTLBs have a better prognosis than do adults. Treatment of comorbidities with SSRIs and CBT was proposed as the most effective therapeutic approach.

A group from the Great Ormond Street Institute of Child Health in London reported preliminary results of a single, 2.5-hour-long psychoeducational group in children with FTLBs ( Pearman *et al*. 2022). Each child selected his own goal-based outcome (GBO), and 31 reported on their GBO after treatment. Self-reported progress on the GBOs was substantial and statistically significant.

### Etiology

**Genetics and epigenetics**

Liao and colleagues performed a transcriptome-wide association study (TWAS) using previously published TS GWAS2 data ( Yu *et al*. 2019) (4819 TS cases and 9488 controls) and CommonMind Consortium and brain tissue panels from GTEx 53 v7 ( Liao *et al*. 2022a). The strongest hit was the *FLT3* gene (which is involved in hematopoiesis, inflammation and immune function), with increased expression in the dorsolateral prefrontal cortex. It was also associated with additional brain regions (cortex, hippocampus, anterior cingulate cortex, frontal cortex, cerebellum, and cerebellar hemispheres). *FLT3* was also supported as the causal gene among the genes in the TWAS region by fine-mapping analysis. Based on a splicing TWAS, the authors identified three additional genes associated with TS ( *MPHOSPH9*, *FIP1L1*, and *CSGALNACT2*), which are also involved in immune processes.

Grotzinger and colleagues ( Grotzinger *et al.* 2022) analyzed publicly available GWAS data from European populations for 11 psychiatric traits, including TS with an aim to investigate their genetic architecture at biobehavioral, functional genomic, and molecular genetic levels. First, they performed genetic correlation and genomic structural equation modeling (SEM) to explore how the studied traits may cluster genetically. TS, anorexia nervosa (AN) and OCD were found to cluster together in one factor. B TS was also part of the Neurodevelopmental disorders cluster, primarily made up of ADHD and ASD, but also including major depressive disorder, post-traumatic stress disorder (PTSD), and problematic alcohol use. The authors also performed additional analyses to identify correlations between the identified clusters and other phenotypes including biobehavioral traits, brain morphology, and circadian activity. They performed cross-disorder meta-analyses of all psychiatric traits, and within each factor to identify shared genetic risk variants. They found one susceptibility locus shared across disorders in the Compulsive Disorders factor), and nine shared loci for the Neurodevelopmental Disorders factor, two of them were new hits; not significant in the individual GWASs included in the analysis.

Tetsos and colleagues performed the largest Tourette Syndrome GWAS meta-analysis to date with a total of 6,133 TS cases and 13,565 controls, including a novel dataset ( Tsetsos *et al*. 2023). They identified a novel genome-wide significance locus on chromosome 5, close to the *NR2F1-AS1* gene. Animal studies have shown that *NR2F1-AS1* is critical in neurodevelopment, and in humans, mutations can cause neurodevelopmental disorders or optic atrophy. The implication of this locus was also supported by analyses combining eQTL and Hi-C (high-throughput chromosome conformation capture) data with the GWAS results. Additional analyses exploring the association of TS polygenic risk score with brain volume data revealed statistically significant associations with right and left thalamus volumes and right putamen volume.

Jain and colleagues used previous TS GWAS summary statistics ( Tsetsos *et al*. 2023) to calculate the TS Polygenic Risk Score (PRS) on individuals in the UK Biobank and then performed a Phenome Wide Association Study (PheWAS) to assess the association of TS genetic risk with a wide range of phenotypes (n = 2242) ( Jain *et al*. 2023). They observed significant associations with 57 traits including anxiety disorder, depression, type 2 diabetes, heart palpitations, and respiratory conditions. They also performed cross-disorder comparisons of the PheWAS results with ADHD, ASD, and OCD. They observed a pattern in which phenotypes associated with TS had a similar direction of effect with ADHD and ASD, but an opposite direction for OCD in most cases except for mental health factors. Additionally, they performed a sex-specific PheWAS, and observed type 2 diabetes and heart palpitations to be significantly associated with TS risk in males but not in females, while diseases of the respiratory system were associated with TS risk in females but not in males.

Ryan and colleagues performed an identity-by-descent (IBD) analysis using Whole Genome Sequencing (WGS) data from 19 individuals from an extended pedigree from a Costa Rican family (17 TS-affected and two controls) ( Ryan *et al*. 2022). Among the implicated TS loci, they observed a rare deleterious missense variation in the *RAPGEF1* gene and two ultra-rare deleterious intronic variants in the *ERBB4* and *IKZF2* genes. All three genes play a role in neurodevelopment.

Ni and colleagues performed a two-sample Mendelian randomization analysis, to explore the causal association between the gut microbiome and 15 psychiatric disorders including TS ( Ni *et al*. 2022). For TS they used the PGC GWAS summary statistics ( Yu *et al*. 2019), and the gut microbiome MiBioGen GWAS summary statistics ( Kurilshikov *et al*. 2021). Consistent with previous literature, they observed a negative causal association of TS with class Bacteroidia and its child taxon, order Bacteroidales.

Mahjani and colleagues analyzed data from a population-based cohort study in Sweden including 993 individuals with OCD, 217 individuals with chronic tic disorders (CTD), and 91 diagnosed with both OCD and CTD ( Mahjani *et al*. 2022). Their aim was to evaluate the distribution of potentially damaging copy number variation (pdCNV) in OCD and CTD patients. Focusing on CTD, they observed 19 pdCNV events in 18 CTD individuals, and three of them were within known genomic disorders (16p13.11 deletion, 15q25.2 deletion, and 17q12 duplication). The 16p13.11 deletion is a locus associated with multiple neurodevelopmental disorders including anxiety disorders, ASD, epilepsy and learning difficulties. Similarly, 15q25.2 was previously reported in individuals with ASD. They also detected 9 pdCNV events in eight individuals diagnosed with both OCD and CTD.

Bae and colleagues analyzed 131 human brains, 19 of which were from donors with TS, to detect somatic mutations through genome-wide sequencing ( Bae *et al*. 2022). When they explored all samples for the presence of somatic mutations on genes related to neuropsychiatric disorders, they did not detect deleterious somatic mutations in TS-related genes. They did however identify a missense mutation in the *ARHGEF6* gene. In the TS cohort they also detected two duplications on chromosome 3 in two different brains present in all bulk samples, suggesting that these occurred during development. Genes in duplicated regions included *RP11-553D4.2*, *SPICE1*, *WDR52* in one sample and *FHIT1* and *U3* in the other.

**Environmental risk factors**

Abdulkadir and colleagues explored whether pregnancy-related risk factors (PRS) (cumulative adverse pregnancy risk score, and maternal anxiety, depression, smoking, and alcohol use) together with genetic factors (TS PRS) ( Yu *et al*. 2019) are associated with the presence of tics in the Avon Longitudinal Study of Parents and Children (ALSPAC) cohort (612 cases and 4,201 controls) ( Abdulkadir *et al*. 2022). Initially, they tested whether each single predictor—PRS and all four pregnancy-related risk factors—were individually associated with the presence of tics in cases versus controls; all predictors except maternal alcohol use were significantly associated with tic presence. Then, they tested the joint effect of TS PRS and each (as well as all) of the pregnancy-related risk factors (except maternal alcohol use). They observed that the “all joint” model explained significantly more variance in the tics presence than the model including only the TS PRS.

**Transient environmental effects on tics**

Iverson and Black summarized research on external factors and internal states that transiently worsen or improve tic severity ( Iverson and Black 2022).

### Pathophysiology

**Electrophysiology**

To better understand the deficit in motor inhibitory control in TS, Indrajeet and colleagues used a recent alternative method (cancellable rise-to-threshold model, CRTT), which distinguishes between proactive (anticipation of a inhibition movement) and reactive (cancellation of a planned unwanted movement) inhibitory control ( Indrajeet *et al*. 2022). They compared 63 patients with TS and 34 HC on an Emotional Stop Signal Task (ESST) and identified robust deficits in TS in reactive control but not in proactive control. The TS group had difficulties in slowing down the speed of movement preparation, which they rectified by their intact ability to postpone the movement.

An EEG study assessing inhibitory control of frontal lobe regions, which are important for motor inhibition in chronic tic disorders, was conducted using a stop signal task ( Zea Vera *et al*. 2022). Right superior frontal gyrus gamma event-related desynchronization (ERD) was elevated in patients with chronic tic disorders during stop preparation. Elevated right superior frontal gyrus gamma ERD correlated with decreased tic severity, suggesting that right superior frontal gyrus gamma ERD may reflect a mechanism of tic suppression. In the future, it is envisioned that electroencephalography might be useful as a biomarker in TS and in understanding the pathophysiology of tics.

TMS was also discussed as a biomarker for tic disorders, including reduced short-interval intracortical inhibition at rest, which suggests a correlation with motor tic severity, shortened cortical silent period duration, increased intracortical facilitation, and decreased motor evoked-potential amplitude ( Jannati *et al*. 2022). Using EEG as a biomarker for comprehensive behavioral intervention (CBIT), a randomized controlled trial was conducted to determine whether EEG coherence during Go/NoGo tasks correlated with CBIT outcomes ( Morand-Beaulieu *et al*. 2022). No association was found between EEG coherence during the Go/No-Go task and changes in tic severity, suggesting that brain processes in the inhibition of Go/No-Go motor responses do not play any role in CBIT.

**Neuroimaging studies**

Baldermann and colleagues reported data from 15 TS patients implanted with thalamic DBS. A positive relationship with tic reduction was observed for functional connectivity between the estimated activated tissue by DBS and the sensorimotor cortex, the bilateral insula and the inferior frontal cortex ( Baldermann *et al*. 2022). A negative relationship was found between the activated tissue and the cerebellum, the temporal and orbitofrontal cortex, and the ventral striatum.

Three publications used MRI to identify pathophysiological abnormalities in TS. The first one ( Bharti *et al*. 2022) highlighted that pure TS, as well as TS associated with OCD (both drug-naïve), were underpinned by increased white matter fractional anisotropy in the corticospinal tract, the anterior thalamic radiations, the inferior longitudinal fasciculus and the corpus callosum, and all correlated with tic severity. The second ( Liao *et al*. 2022b) also focused on drug-naïve children with TS, assessed alterations in brain network topology. Some network metrics were abnormal at the global level ( *i.e.*, increased global efficiency and decreased path length), and they also showed some nodal changes, especially in the cortico-striato-thalamo-cortical circuit ( *i.e.*, SMA, caudate nucleus, thalamus, superior parietal gyrus, posterior cingulate cortex). This study was especially relevant since the authors used brain metrics which are rarely investigated. The third study focused on gyral abnormalities ( McCann *et al*. 2022). They identified that younger patients with TS (children) had increased surface curvature in the frontal cortex (opercular and triangular parts of the inferior frontal cortex), while older patients (adolescents) had increased grey matter volume in the cerebellum, the precentral cortex and the primary motor cortex.

Another interesting work was published last year, using different statistical models to classify BOLD resting state fMRI of patients with TS and healthy controls ( Xin *et al*. 2022). The model that achieved the highest accuracy (multivariate non-linear model, accuracy 94%, sensitivity 96% and specificity 92%) was especially based on changes observed in the frontal cortex (superior, medial and middle frontal gyrus, SMA, pre- and postcentral cortex), basal ganglia (putamen, thalamus and caudate nucleus) and the cerebellum. In the future, this kind of work could lead to specific tools which might contribute to the diagnosis of TS.

From a more cognitive viewpoint, three studies linked specific TS symptoms to brain alterations. The first one investigated the relation between the structural connectivity of several basal ganglia (caudate nucleus, putamen, nucleus accumbens, subthalamic nucleus and the medial subthalamic region) and the cortex with anxiety and impulsivity in TS ( Temiz *et al*. 2022). They found hyperconnectivity in TS patients between the left medial subthalamic region and the insula, entorhinal and temporal cortex. Moreover, the connectivity between the subthalamic nucleus and the left insula was positively related to impulsivity and anxiety scores, measured respectively with the BIS-11 and the STAI. The second study focused on the role of GABA as related to the urge to tic and several measures of cortical inhibition obtained with TMS ( Larsh *et al*. 2022). The authors found that severe urges were negatively correlated to cortical excitability and long-interval cortical inhibition in the primary motor cortex. However, they also found that more severe tics were positively correlated with both of these measures. Last, they found that GABA in the right SMA (cortical excitability and long-interval cortical inhibition) was changed in TS patients compared to healthy controls. Altogether, they concluded that changes in the primary motor cortex were modulated by GABA within the SMA and could reflect compensatory mechanisms. The third study focused on error-related negativity measured by MEG ( Metzlaff *et al*. 2022). This measure is known to reflect processes of performance monitoring, to be increased during error processing and conflicting response, and to be related to the dopaminergic system and prefrontal cortex activity. In a small sample of adult patients without any medication nor comorbidities (n = 8) performing a Go/No-Go task, the authors showed a significant interaction between groups (TS vs. healthy controls) and response (correct vs. error). The authors explained this difference by suggesting that TS patients processed all their responses as erroneous, which means that TS patients had an altered performance monitoring.

A fascinating preliminary report used a clever experimental design to observe regional brain activity (fMRI BOLD) while people with and without TS either chose themselves whether or not to act on Go/No-Go task or followed visually displayed “Go” or “No-Go” instructions in the same situation ( Rae *et al*. 2022). The authors found that activity patterns in the pre-supplementary motor area (preSMA) differed between groups, such that its activity was similar across all task conditions in TS, whereas in control participants it differed by locus of decision-making and by action *vs.* inhibition. The authors interpret the results as suggesting that brain activity in the preSMA region codes action categories more rigidly, which may explain the sense of effort required to suppress tics voluntarily and the sense of agency that differentiates tics from many other movement disorders.

### Treatment

**Psychological interventions**

Behavior therapy (BT) is recommended as the first-line intervention for TS/CTD in treatment guidelines published by the American Academy of Neurology (AAN) ( Pringsheim *et al*. 2019) and the European Society for the Study of Tourette Syndrome (ESSTS) ( Müller-Vahl *et al*. 2021). Two types of BT are recommended. The first is Habit Reversal Training (HRT) and its extended package Comprehensive Behavioral Intervention for Tics (CBIT), which has the stronger evidence base. The second recommended BT intervention, Exposure and Response Prevention (ERP) has comparatively less support but is especially popular among clinicians and researchers in Europe ( Andrén *et al*. 2022b).

Remote delivery of BT has become increasingly popular within the last years as a way to make BT more accessible to healthcare seeking individuals with TS/CTD. Several new studies on remote delivery of BT were published in 2022. The largest study by far was conducted in Sweden by Andrén and colleagues ( Andrén *et al*. 2022a), in which 221 young individuals were randomized to therapist-supported, internet-delivered ERP or therapist-supported, internet-delivered psychoeducation (comparator). The results showed that both groups improved on the primary outcome (tic severity as measured by the Yale Global Tic Severity Scale’s Total Tic Score [YGTSS-TTS]) from baseline to the primary endpoint (three months post-treatment). YGTSS-TTS reductions were 6.1 points (27%) in the ERP group and 5.3 points (23%) in the comparator. Contrary to the similar ORBIT study conducted in the UK ( Hollis *et al*. 2021), there was no significant interaction of group and time on the primary outcome. Despite this, treatment response rates at the three-month follow-up were identical in both studies, with significantly more treatment responders in ERP (47%) than in the comparator (29%). The authors concluded that both internet-delivered interventions may be efficacious for young individuals with TS/CTD. Long-term follow-up data will appear in a future publication.

Internet-delivered BT has also recently been evaluated in Israel. Originally published online in 2020, Rachamim and colleagues compared Internet delivered CBIT to a wait list in a randomized controlled trial (RCT) of 41 youth with TS/CTD ( Rachamim *et al*. 2022). The results showed that Internet delivered CBIT was feasible to implement and superior to waitlist. In a new analysis of the same data ( Rachamim *et al*. 2021), the authors focused on 27 treatment completers with comorbid attention deficit hyperactivity disorder (ADHD; n = 16) or comorbid obsessive-compulsive disorder (OCD; n = 11). This new analysis showed that, as in the complete sample, tic severity (YGTSS-TTS) improved in both the ADHD and the OCD groups, although the OCD group improved significantly less than participants without comorbid OCD.

Another way of delivering BT remotely is through videoconferencing, a format which increased in popularity worldwide during the COVID-19 pandemic. In an Italian RCT conducted by Prato and colleagues, 40 youth with TS were randomized to BT (HRT or ERP) delivered face-to-face at a clinic or remotely via videoconference ( Prato *et al*. 2022). Participants improved on the YGTSS-TTS in both groups, in line with previous studies ( Himle *et al*. 2012; Ricketts *et al*. 2016). However, contrary to the authors’ claim, this did not indicate that the two interventions were equally efficacious, since the study lacked a pre-defined non-inferiority aim. The videoconference format was also partly used in a naturalistic study of ERP conducted at a TS/CTD specialist clinic in Denmark. In this study by Sørensen and colleagues ( Sørensen *et al*. 2023a, 2023b), 116 youth received ERP (either face-to-face [n = 72] or via videoconference [n = 44]) and were followed up one-year post-treatment. The study showed significant short- and long-term tic severity reductions (YGTSS-TTS) in both groups, with no significant between-group differences. Participants who completed the planned ERP sessions or discontinued early due to a satisfactory tic reduction, improved significantly more than participants who dropped out due to lack of motivation. Firm conclusions are limited by the open design, but the study overall provides support for both face-to-face and videoconference delivery of ERP.

Another way to make BT more accessible is the group format, where simultaneous treatment of several individuals by one therapist may save therapist resources. Based in the Republic of Korea, Kang and colleagues conducted a non-randomized controlled study (n = 30) comparing group CBIT (n = 18) to a supportive psychotherapy and psychoeducational control condition (n = 12) ( Kang *et al*. 2022). Overall, the baseline TS/CTD severity of the sample was mild. The CBIT group showed modest improvements, with the clearest result being superiority over the comparator in reducing tic-related impairment. The study provided some preliminary support to the feasibility of providing CBIT in a group format in this Korean context. Inoue and colleagues evaluated group CBIT in a case series (n = 3) in Japan ( Inoue *et al*. 2022). In this study, group CBIT was delivered via videoconference software, to further increase accessibility of BT to individuals in the region. The results showed an average tic severity reduction (YGTSS-TTS) of seven points from baseline to post-treatment. Overall, the treatment was concluded to be feasible, acceptable, and promising for further evaluation.

A few studies on face-to-face CBIT were also published in 2022. In a US study, Greenberg and colleagues evaluated a modified CBIT intervention, which also included the treatment of comorbid ADHD and psychosocial impairment from both TS/CTD and ADHD ( Greenberg *et al*. 2023). Seventeen young participants with both TS/CTD and ADHD were randomized to modified CBIT (n = 9) or a traditional CBIT comparator (n = 8). The results showed significant improvements in tic severity, tic-related impairment, and ADHD severity for both groups, but the study was likely underpowered to detect between-group differences. Overall, the results indicated feasibility and acceptability for this modified treatment for youth with TS/CTD and ADHD. In a Chinese RCT, Xu and colleagues recruited 37 youth with TS/CTD to compare face-to-face CBIT (n = 12), face-to-face CBIT combined with pharmacotherapy (n = 10), and pharmacotherapy alone (n = 15) ( Xu *et al*. 2022). Tic severity (YGTSS-TTS) improved for all three groups between baseline and post-treatment. Although the approach of comparing BT and pharmacotherapy is relatively novel for the TS/CTD field, the study lacked sufficient power for between-group comparisons.

Lastly, interest in investigating the underlying working mechanisms of BT has increased in recent years. In a study of 80 adults with TS, Ramsey and colleagues ( Ramsey *et al*. 2022) used structural equation modeling (SEM) to examine the distress provoked by the PU in patients with tics. They concluded that higher levels of PU intolerance predicted greater levels of tic severity and tic-related impairment. This result highlights a potential clinical implication of targeting the concept of PU intolerance, rather than for instance PU strength, in BT.

In an RCT including 53 youth, McGuire and colleagues ( McGuire *et al*. 2022) investigated the relationship between several pre-selected cognitive control processes and face-to-face CBIT outcomes. The results showed that only one of the investigated processes—baseline inhibition/switching (as measured by the D-KEFS Color Word Interference Test)—predicted post-treatment tic severity. Interestingly, Gur and colleagues ( Gur *et al*. 2022) studied cognitive inhibition and emotion regulation before and after CBIT group therapy. Fifty-five participants aged 8–15 years with tic disorders were randomly assigned to the CBIT group or the Educational Intervention group and compared on tests of cognitive inhibition and emotion regulation strategies. Their results showed an increase in cognitive reappraisal in the CBIT group only, which was associated with higher intellectual ability. This study raises the possibility that CBIT contributes beyond tic control to cognitive and emotional regulation.

Morand-Beaulieu and colleagues ( Morand-Beaulieu *et al*. 2022) conducted a similar RCT (n = 32), but focused instead on underlying brain mechanisms, particularly EEG coherence. Face-to-face CBIT was superior to the treatment-as-usual comparator, but EEG coherence during a Go/No-Go task was not associated with the tic severity outcome. In a small open study by Eapen and colleagues (n = 17) ( Eapen *et al*. 2022), tic severity significantly improved following face-to-face CBIT. Further, the study showed preliminary support for neurophysiologic changes in cortical inhibition as a potential underlying working mechanism. To summarize, considering that CBIT is a package of numerous behavioral exercises, several underlying mechanisms may be part of explaining how and why the treatment works.

**Pharmacological studies**

A comprehensive and systematic review of pharmacological treatments for patients with TS was provided by Farhat and colleagues ( Farhat *et al*. 2023), confirming that antipsychotic drugs are the most efficacious intervention for tics, followed by α-2 agonists.

Abi-Jaoude and colleagues compared the efficacy and tolerability of single doses of three vaporized medical cannabis products and placebo in reducing tics in adults with GTS ( Abi-Jaoude *et al*. 2022). Each participant received a vaporized single 0.25 g dose of Δ ^9^-tetrahydrocannabinol (THC) 10%, cannabidiol (CBD) 13%, THC/CBD 9%/9%, and placebo at 2-week intervals. There were no statistically significant differences in tic severity for any of the cannabis-based medicine in primary outcome, but THC 10% was significantly better than placebo on the secondary outcome measures including assessments for premonitory urge, stress, and clinical global impression.

An interesting first study randomized 34 children with TS/CTD 1:1 to either a combination of the amino acid L-theanine 200 mg/d and low-dose vitamin B6 2.8 mg/d, or to eight sessions of psychoeducation, for two months ( Rizzo *et al.* 2022). There was no blinding, but the results were interesting: in the medication group, 71% were responders (YGTSS total tic score decreased by at least 30%), compared to 18% of the control group. The authors appropriately note that these results need confirmation in a larger trial with blinded assessment and matching placebo pills.

**Neurosurgery**

An elegant study by Ganos and colleagues ( Ganos *et al*. 2022) offers new insights regarding our knowledge of brain networks related to tics and the implications for deep brain stimulation (DBS). They studied 22 patients with secondary tics caused by various types of brain lesions and employed lesion network mapping to identify a common neural network implicated in tic generation. Their methodological approach combined: (i) a comparison of brain lesions which induced tics (n = 22) to control brain lesions which did not induce tics (n = 717); (ii) they built a functional lesion network using healthy subjects’ fMRI (n = 1000) and using the lesion location they found in the first step as seed; and (iii) they assessed the utility of this lesion network to predict tics decrease after thalamic deep brain stimulation surgery on patients with TS (n = 30). They found that despite the very varied brain locations of tic-inducing lesions, regions functionally connected to these lesions mapped to a common network encompassing the insular cortices, the cingulate gyrus, the striatum, the globus pallidus internus (GPi) and the cerebellum. The connectivity of the anterior striatum was significantly more associated with tics compared to lesions inducing other types of movement disorders. They then collected data from 30 patients with TS who had undergone thalamic or pallidal neurostimulation and found that the overlap between the site of neurostimulation and the lesion network map was predictive of tic improvement. Zouki and colleagues presented a preliminary report of a similar analysis, using as seeds both structural abnormalities in TS from neuroimaging studies and reported lesions inducing tics ( Zouki *et al*. 2022). They found that network connections converged on thalamus, striatum, globus pallidus pars externa, and the occipital lobe.

Several targets have been used for DBS in TS, and the question of the optimal target is still debated. Dai and colleagues report the outcomes of dorsal (sensorimotor) STN DBS in 10 patients with TS and showed an overall reduction of 62.9% of tics at six months and 58.8% at 12 months, with an improvement in quality-of-life measures and OCD ( Dai *et al*. 2022). Another large retrospective study included 61 patients with refractory TS who were implanted in the posteroventral GPi with an overall improvement of the YGTSS of 58.1% at last follow-up (30 to 130 months) ( Cui *et al*. 2022). As previously reported in other studies, there was an important heterogeneity in clinical response, as five patients were very much improved and five had no effect of DBS. Another case series of seven patients operated in the posteroventral GPi showed a mild effect on tics and OCD but a more substantial effect on depressive symptoms ( Liu *et al*. 2022). Five patients who were operated in the anteromedial GPi and had an overall improvement of 65% of the YGTSS and 75% reduction of the Y-BOCS. Two patients were free of medication post-operatively.

The programming paradigm of DBS in TS has been seldom studied and programming remains empirical. In previous studies, patients were mostly treated with high frequency stimulation (130 Hz or above). Lower frequency stimulation could prolong the battery life and be better tolerated. Sun and colleagues studied the effect of low frequency (65Hz) stimulation of the GPi and showed a median reduction of the YGTSS of 58.2% at one year, and an improvement of YBOCS of 48.4%, which is in line with reports of DBS at higher frequencies ( Sun *et al*. 2022)

The outcomes of DBS in GTS patients under 18 years are increasingly documented. Srinivas and colleagues reported the case of a 14-year-old boy with severe tics, self-mutilation and comorbid OCD and ADHD who underwent DBS of the anteromedial GPi. He had substantial improvement of both tics and OCD at six months ( Srinivas *et al*. 2022).

Ablative surgery can be an alternative to DBS with the advantage of lower invasivity but with the caveats of being irreversible and less adjustable. Wang *et al*. report the outcomes of four patients who had radiofrequency thermo-ablative lesions of several targets, eight patients with DBS (GPi in the majority), and one patient with combined DBS and ablation ( Wang *et al*. 2022). They report overall good outcomes in their group of patients who had undergone ablative surgery with a 53.3% reduction in global YGTSS score but did not compare the outcomes of patients treated with DBS to patients with ablative surgery. Notably, two patients who received ablative surgery developed complications such as apathy, urinary incontinence, dysphagia and stereotypic movements.

Lin and colleagues published a meta-analysis comparing the efficacy of DBS, repetitive transcranial magnetic stimulation (rTMS) and BT ( Lin *et al*. 2022). The showed that the three treatments significantly improved tics. Further analyses found that DBS was the most effective in reducing tics, followed by BT and rTMS. Conversely, rTMS was the most efficient to reduce associated OCD, followed by DBS and BT. These conclusions have to be tempered by the fact that patients included in the DBS and rTMS studies did not have the same profile: the patients referred to DBS had more severe tics and patients referred to rTMS had more severe psychiatric comorbidities.

**Other treatments**

Following up on their fascinating report of median nerve stimulation (MNS) treatment for tics in TS ( Morera Maiquez *et al*. 2020), Houlgreave and colleagues performed an MEG study showing contralateral hemisphere cortical effects of rhythmic (but not arrhythmic) MNS at 12Hz or 20Hz ( Houlgreave *et al*. 2022). In an open-label study of MNS in 31 adults and older teenagers with TS, participants reported treatment benefit and good tolerability, both in the moment via brief online surveys and at the end of the study ( Iverson *et al*. 22AD; Iverson *et al*. 2023).

Transcranial magnetic stimulation (TMS) as a treatment modality for tics has been discussed in several recent reviews. Repetitive TMS has been shown to improve tic symptoms and tic comorbidities, and its safety has been confirmed ( Bejenaru and Malhi 2022). Other review articles have discussed not only rTMS but also other electrophysiological modalities such as transcranial direct current stimulation (tDCS), peripheral nerve stimulation, and cranial electrotherapeutic stimulation (CES) for the treatment of tics ( Frey and Malaty 2022) ( Dyke *et al*. 2022). In contrast to repetitive TMS, tDCS, which works by applying a constant low current to electrodes attached directly to the scalp, is inexpensive, portable, and easy to implement. To date, results have been mixed and inconclusive, as many studies have been open-label designs. Vagus nerve stimulation (VNS) treatment has also been reported to improve tic symptoms, but it is still unknown how VNS affects tic symptoms. CES is a small, portable device that stimulates the brain with a weak electric current and is being studied for its effectiveness in treating tic disorders.

### Tics, family and society

In a large Swedish cohort study with more than 13 million individuals and almost 7,800 individuals with TS or CTD, it was found that persons with TS or CTD did have an increased risk of experiencing any violent assault or violent and nonviolent crime convictions ( Mataix-Cols *et al*. 2022). The presence of comorbid ADHD and substance use disorders increased the risk. Similarly, more than half of the children with a TS diagnosis included from than 2016–2017 National Survey of Children’s Health data on children from 6 to 17 years, had experienced bullying victimization, and around 20% had perpetrated bullying; both are substantially more frequent than in children without TS ( Charania *et al*. 2022). Martino and colleagues thoughtfully discuss some of the difficulties that arise in interpreting these reports ( Martino *et al*. 2022).

The Tourette Association of America (TAA) conducted a web-based anonymous survey of adults with TS (n = 601) and parents of children with TS (n = 593) ( Tourette Association of America 2022). The sample was not ascertained on a population basis but is likely to represent reasonably well patients at referral centers or engaged in the American TS community. Many of the findings were rather dramatic: for instance, 23% of children and 48% of adults reported that they had considered suicide at some point in their lives, and 10% of children had attempted suicide in the past year. Over two thirds reported they had been discriminated against because of tics. Parents reported substantial impact: 15% of them lost their job or had to reduce their work hours to care for their child with tics, and 5% had to move due to financial stressors from managing tics. On the positive side, it was reported that half of children were diagnosed within a year of symptom onset (much earlier than in previous studies), and three-fourths of children had a legally binding individualized education plan.

A longitudinal cohort study showed that academic achievement was decreased in children with TS and the severity of comorbidities was shown to have greater influence on education than did tic severity ( Lund *et al*. 2022).

Atkinson-Clement and colleagues examined the consequences of TS on adolescents’ daily living by using a text mining approach ( Atkinson-Clement *et al*. 2022a). Social stigma was shown to be the most common issue faced by patients with TS. Tic severity had an especially an impact on daily life at school while comorbidities mostly were related to social daily living and risk of depression.

A survey by a pharmaceutical company investigated the educational needs of neurologists, psychiatrists, and caregivers ( Stacy *et al*. 2022). Among other findings, neurologists and psychiatrists differed in their approach to diagnosis and treatment, and parents and physicians differed on the locus of treatment decision-making and on the approach to treating tics that are only “slightly bothersome.”

Mealtimes and feeding are known to be problematic in children with TS, both in and out of the home. Using a semi-structured interview, the full scope of difficulties was captured, and potential solutions offered ( Bamigbade *et al*. 2022).

Stofleth and Parks conducted semi-structured interviews in 18 adults with TS ( Stofleth and Parks 2022). The aim was to assess how others respond to TS behaviors and how these are misinterpreted in interpersonal interactions, using thematic analysis. Unsurprisingly, all participants received unwanted attention regarding their tics, which could be subdivided into six subthemes. Also, three types of misunderstanding in interpersonal interactions were identified. This article is a rewarding read with many concrete examples.

Lee and colleagues explored the role of self-esteem in the relationship between psychosocial stress and social adjustment among adolescents with TS ( Lee *et al*. 2022). The authors found that self-esteem fully mediates the relationship between their psychosocial stress and social adjustment, while comorbidities moderate the relationship between self-esteem and social adjustment.

Ricketts and colleagues described impairment in academic, interpersonal, recreational, and family financial or occupational domains across children in three groups: with GTS, ADHD and both disorders. Children with ADHD (with or without TS) experienced a greater degree of impairment in overall school performance, writing, and mathematics, relative to children with TS alone. More children with TS and ADHD had problematic handwriting relative to children with ADHD alone. More children with TS and ADHD had problematic interpersonal relationships relative to those with ADHD alone. Children with TS and ADHD had higher mean impairment across domains than children with either TS or ADHD ( Ricketts *et al*. 2022b).

Soós and colleagues investigated the online support available for tic disorders by an inductive thematic analysis of posts and comments. They suggest that online support communities might be valuable in sharing and gaining information on tics from other patients ( Soós *et al*. 2022).

Hall and colleagues examined the impact of the COVID-19 pandemic on individuals with tics. They compared YGTSS pre- and post-pandemic in 112 children and adolescents with tics. There were no significant differences in tic symptom or severity between participants who were assessed before and during COVID-19 ( Hall *et al*. 2022).

Similarly, Termine and colleagues investigated the burden of the COVID-19 pandemic and lockdown on individuals with tics. The authors included 49 children with tics and 245 matched controls who were asked to provide information about lockdown-related changes to daily activities. More than half of patients reported perceived changes in tic severity, restlessness and irritability during the pandemic ( Termine *et al*. 2022).

### Additional sources

In 2022, the **International Review of Movement Disorders** book series published a large work on TS, divided into two volumes ( Lavoie and Cavanna 2022a, 2022b). The book includes up-to-date contributions from a Who’s Who of TS research, from history to neurobiology.

Similarly comprehensive in scope, the second edition of **Tourette Syndrome** also appeared during 2022, now edited by Davide Martino in addition to James Leckman ( Martino and Leckman 2022).

## Conclusions

This year, FTLBs were again a focus of research and concern for clinicians. It is hoped that this wave of functional patients will eventually abate as the COVID-19 pandemic recedes, but this remains to be seen: alternative sources of anxiety abound in today’s world and the sometimes toxic influence of social media will likely persist if not increase. On a positive note, studies related to behavioral therapies in TS/CTD are plentiful and of excellent quality, which is cause for hope in the treatment of tics and comorbidities. In contrast, no significant pharmacological developments were reported in 2022, but hopefully this is the quiet before a productive storm. Finally, the entry of non-invasive brain stimulation into the clinic looms large and is encouraging, either as a stand-alone treatment or combined with behavioral and pharmacotherapy.

## Data Availability

No data are associated with this article.
