# Supplementary material for: Tourette syndrome research highlights from 2022
Source: F1000Res. 2023 Oct 23;12:826. Originally published 2023 Jul 13. [Version 2] doi: 10.12688/f1000research.135702.2 (PMC10483181; doi:10.12688/f1000research.135702.2)
Supplement: Peer review history

# Reviewer report: Reviewer response for version 2

**DOI:** 10.5256/f1000research.157104.r217617

**Published:** 2023-11-09

**Authors:** Erica L. Greenberg

**Affiliations:** 1 Department of Psychiatry, Massachusetts General Hospital, Boston, MA, USA

No further comments, I appreciate the edits!

Is the review written in accessible language?

Yes

Are all factual statements correct and adequately supported by citations?

Yes

Are the conclusions drawn appropriate in the context of the current research literature?

Yes

Is the topic of the review discussed comprehensively in the context of the current literature?

Yes

Reviewer Expertise:

Tourette syndrome, OCD, child and adolescent psychiatry

I confirm that I have read this submission and believe that I have an appropriate level of expertise to confirm that it is of an acceptable scientific standard.

# Reviewer report: Reviewer response for version 1

**DOI:** 10.5256/f1000research.148833.r186883

**Published:** 2023-09-06

**Authors:** Jeremy Stern

**Affiliations:** 1 Department of Neurology, St George's, University of London, London, UK

As introduced by the authors this is the ninth such publication, providing a very useful and by now customary (not to mention awaited) round-up of the field, from the clinical and therapeutic to scientific, including taking in selected reviews.

 There are regular published reviews of Tourette syndrome both in the round and more focussed areas and some are rather substantial. The purpose of this admirably concise article cannot be to provide the history and full context of each research direction, rather it serves to give a helpful snapshot of publishing in the whole area from the last year. It manages to be comprehensive in important themes, divided into 5 categories, but not over inclusive in data. It is balanced and impartial. It is inevitable that reading it will improve one's breadth of knowledge.

 For readers less familiar with the area it may be useful to start with the conclusions which signpost  areas of the last year that are attracting the most attention, both in the research and patient communities. A striking absence is also highlighted, the paucity of recent new pharmacological work.

 I note one paper is cited twice in both the pathophysiology and treatment sections in fairly similar terms (Morand-Beaulieu et al 2022), but undoubtedly does deserve inclusion in the review.

Is the review written in accessible language?

Yes

Are all factual statements correct and adequately supported by citations?

Yes

Are the conclusions drawn appropriate in the context of the current research literature?

Yes

Is the topic of the review discussed comprehensively in the context of the current literature?

Yes

Reviewer Expertise:

Tourette syndrome.

I confirm that I have read this submission and believe that I have an appropriate level of expertise to confirm that it is of an acceptable scientific standard.

# Reviewer report: Reviewer response for version 1

**DOI:** 10.5256/f1000research.148833.r186880

**Published:** 2023-09-06

**Authors:** Erica L. Greenberg

**Affiliations:** 1 Department of Psychiatry, Massachusetts General Hospital, Boston, MA, USA

Thank you for the opportunity to review this important manuscript that summarizes key additions in the literature to the field of Tourette over the last year. The review is extensive, thorough, and covers a multitude of areas pertinent to TS, including pharmacology, genetics, behavioral treatments, epidemiology, etc. 

 It is very well organized, and I recommend acceptance with minor changes. 

 Most of the changes are regarding small changes in the results (see below), and a handful of changes to the Conclusion.

 Results: for the paragraph starting...

- " *Bazaibal-Carvallo and colleagues...*" - it would help to describe what " *self-inflicted damage*" is - and how that differs from SIB
-  " *A huge electronic...*" Is it appropriate to say the " *risk of atopy was higher in children*" ? I imagine it would be more appropriate (unless methodology was described further) to say " *rates of atopy were higher..*."
- " *Rickets and colleagues*" - it would help to comment on what the authors mean by "important predictors" - significance? magnitude? what p-value if that's the correct statistic?
- " *Li and colleagues*" - looks like PU isn't defined until the below paragraph - would write out premonitory urges in this one
- *"Langelage and colleagues*" - it's hard to follow what the author means by "positive association between urge and tic" and "reverse association"
- In the comorbidities section, given that there are so many articles focusing on sleep, I would make sleep its own category
- " *Ricketts and colleagues published...*" - Would recommend defining chronotype
- " *Another study focused on decision-making processes*" - I had trouble following this summary, and would recommend re-writing/re-phrasing for clarity purposes
- " *Fremer and colleagues*" - was confused what the authors were referring to with "structural deficits" and "unconscious intrapsychic conflicts" for that matter
- " *Trau and colleagues*" - Starting " *Similarly, the Calgary group tested which symptoms" through "self-injurious actions*" would benefit from being moved to the below paragraph as those findings are more consistent with teh subsequent paragraph.
- " *Grotzinger and colleagues...*" - Difficulty understanding this summary, would re-write/edit, especially from "TS, anorexia nervosa" through "alcohol use." Seems like the last sentence of the paragraph contradicts an earlier sentence in the paragraph
- " *Abdulkadir and colleagues*" - Clarify what PRS means
- " *A fascinating preliminary...*" - Should the authors say "in TS" following the clause " *across all task conditions*" ?
- " *Remote delivery of BT*" - starting with " *However, treatment response rates...*" - paragraph became hard to follow
- " *A few studies on face-to-face*" would change "conducted" to "published" since the studies weren't conducted in 2022.
- " *Lastly, interest in investigating*" ... would change "later" to "recent;" would make use of PU vs premonitory urges consistent; would clarify "instance urge severity"
- " *Abi-Jaoude and colleagues*" - would help to list some secondary outcome measures
- " *Lin and colleagues*" - unclear how the behavioral therapy performed in comparison
- " *The Tourette Association of America*" - would make sure to say "reported" when describing some of the self-reported statistics 
- " *A longitudinal clinical cohort study...*" - would edit for grammar
- " *Atkinson-Clement and colleagues*" - grammar bingeing " *Severity of tics...*"

Conclusions:

- It actually seemed like the focus on FTLBs this year was a lot less than the prior year
-  Would change wording of " *this wave of functional patients will eventually abate*" as is sounds more negatively judgmental than I believe the authors intended, and additionally, really wasn't a large focus on the summaries this year. Similarly, I would strongly recommend eliminating the sentence referring to " *toxic influence of social media*" as that was not a focus of many/any articles that were reviewed, and it sounds unduly biased against social media when the literature is less clear. 

Final thought is that I would recommend considering a more standardized approach to capturing the array of articles that are published each year, as it is likely that many fall through the cracks for a variety of reasons.

Is the review written in accessible language?

Yes

Are all factual statements correct and adequately supported by citations?

Yes

Are the conclusions drawn appropriate in the context of the current research literature?

Yes

Is the topic of the review discussed comprehensively in the context of the current literature?

Yes

Reviewer Expertise:

Tourette syndrome, OCD, child and adolescent psychiatry

I confirm that I have read this submission and believe that I have an appropriate level of expertise to confirm that it is of an acceptable scientific standard, however I have significant reservations, as outlined above.

# Reviewer report: Reviewer response for version 1

**DOI:** 10.5256/f1000research.148833.r186879

**Published:** 2023-07-18

**Authors:** James F. Leckman

**Affiliations:** 1 Child Psychiatry, Psychiatry, Psychology and Pediatrics, Yale Child Study Center, Yale University, New Haven, Connecticut, USA

This team of scholars and practitioners have once again provided a comprehensive summary of the scientific articles published in 2022 regarding Tourette syndrome (TS) and related disorders. 

 This article will be a valuable resource for clinicians and researchers interested in this topic. For example, I am in the midst of preparing a chapter on Tic Disorders for the 7th edition of the Rutter Textbook on Child and Adolescent Psychiatry, and this article has introduced me to a few recent scientific findings that I was unaware of.

 I was a bit disappointed that there was no mention of the use of Traditional Chinese Herbal Medicines that are currently being used to treat TS. Given that there is strong evidence supporting use of the 5-Lin granule (TSupport) in treating tic disorders, my colleagues and I are about to start a multisite clinical trial in the US. However, since there were no relevant articles published on this topic in 2022, it is reasonable that this emerging area of research was not mentioned in the review.

Is the review written in accessible language?

Yes

Are all factual statements correct and adequately supported by citations?

Yes

Are the conclusions drawn appropriate in the context of the current research literature?

Yes

Is the topic of the review discussed comprehensively in the context of the current literature?

Yes

Reviewer Expertise:

I am actively involved the care of individuals wit TS, and with my colleagues at Yale we are conducting a number of research studies.

I confirm that I have read this submission and believe that I have an appropriate level of expertise to confirm that it is of an acceptable scientific standard.

## References

[1] : Traditional Chinese Medicine Strategy for Patients with Tourette Syndrome Based on Clinical Efficacy and Safety: A Meta-Analysis of 47 Randomized Controlled Trials. *Biomed Res Int* .2021;2021: 10.1155/2021/6630598 6630598 10.1155/2021/6630598 33778073PMC7977981
